# *In vitro* and *in vivo* antischistosomal activity of ferroquine derivatives

**DOI:** 10.1186/1756-3305-7-424

**Published:** 2014-09-04

**Authors:** Jennifer Keiser, Mireille Vargas, Riccardo Rubbiani, Gilles Gasser, Christophe Biot

**Affiliations:** Department of Medical Parasitology and Infection Biology, Swiss Tropical and Public Health Institute, Basel, Switzerland; University of Basel, Basel, Switzerland; Department of Chemistry, University of Zurich, Winterthurerstrasse 190, CH-8057 Zurich, Switzerland; Université Lille 1, Unité de Glycobiologie Structurale et Fonctionnelle UGSF, F-59650 Villeneuve d’Ascq, France; CNRS, UMR 8576, F-59650 Villeneuve d’Ascq, France

**Keywords:** *Schistosoma mansoni*, Ferroquine, Chloroquine, Ruthenoquine, Hydroxyl-ferroquine, *In vitro*, *In vivo*

## Abstract

**Background:**

Schistosomiasis is a neglected tropical disease and drug-repurposing is a useful strategy to fill its exhausted drug development pipeline. The ferrocenyl analogue of chloroquine, ferroquine, is an antimalarial in late-stage drug development. The aim of the present work was to study the antischistosomal activity of ferroquine against *Schistosoma mansoni* adult worms and newly transformed schistosomula (NTS) *in vitro* and *in vivo*. Hydroxyl-ferroquine and ruthenoquine were included to study the potential role of reactive oxygen species in the antischistosomal activity. Chloroquine and mefloquine, the later described for its antischistosomal properties, served as comparators.

**Findings:**

All metal complexes were shown to be moderately cytotoxic on human cervix HeLa cancer cells and human fetal lung fibroblasts MRC-5. 72 hours post-incubation NTS exposed to 33.3 µM ruthenoquine had died, while ferroquine and hydroxyl-ferroquine treated worms were strongly affected. No activity was observed treating NTS with chloroquine at 33.3 µM. Incubation of adult *S. mansoni* with 33.3 µM of the organometallic derivatives were highly affected in viability but were still alive 72 hours post-incubation. Mefloquine showed the highest activity against NTS and adult *S. mansoni*. Low total worm burden reductions of 0-36% were observed following oral administration of 200–800 mg/kg of the ferroquine derivatives to *S. mansoni*-infected mice.

**Conclusions:**

The organometallic compounds evaluated in this study revealed moderate *in vitro* activity against both larval and adult stages of *S. mansoni* but low *in vivo* activity. No correlation can be drawn between the antimalarial and antischistosomal activity of chloroquine analogues and oxidative shock does not seem to play a role in the activity of these compounds against *S. mansoni.*

## Background

Schistosomiasis is a neglected tropical disease, which causes a considerable public health burden [1]. Millions of people require treatment each year to prevent the considerable health effects of schistosomiasis. For example, in 2012 it has been estimated that 249 million people should be treated in preventive chemotherapy programs [2]. Yet, treatment and control of schistosomiasis relies on a single drug, namely praziquantel [1, 3, 4].

**Figure 1:**
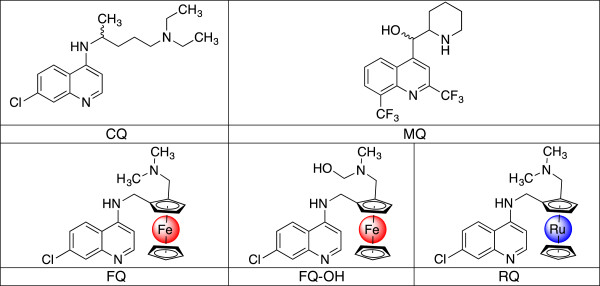
**Chemical structures of chloroquine (CQ), mefloquine (MQ), ferroquine (FQ), hydroxyl-ferroquine (FQ-OH) and ruthenoquine (RQ).**

Research on novel antischistosomal drugs is nearly exclusively driven by academic groups with a strong focus on phenotypic approaches, since protein targets are almost unknown [5]. Over the past years, libraries consisting of existing drugs, so called drug-repurposing, were investigated in order to fill the exhausted drug development pipeline for schistosomiasis [6]. Drug repurposing circumvents the high cost of drug discovery and development, the high failure rates and the long duration to develop novel treatments by finding new uses for compounds other than those they were initially intended to treat [7]. Antimalarials, in particular, have been well studied, which is not surprising since both *Plasmodium* and schistosomes degrade hemoglobin [4]. For example, the excellent antischistosomal activity of mefloquine (MQ, Figure 1) was demonstrated in *S. haematobium*, *S. japonicum* and *S. mansoni* rodent models [8]. The promising results obtained with MQ in laboratory studies were followed up in clinical trials in the past years [9, 10]. Over the last decade, there has been increasing interest in metal-based drugs and numerous metal complexes have been synthesized and evaluated as antimalarial agents [11]. The most promising candidate was undoubtedly the ferrocenyl analogue of chloroquine (CQ, Figure 1), ferroquine (FQ, Figure 1). FQ reached two different Phase IIb clinical trials, in combination with artesunate or OZ439, for the treatment of uncomplicated *Plasmodium falciparum* malaria (http://www.mmv.org/research-development/project-portfolio/oz439fq). FQ has a specific parasiticidal effect on *Plasmodium* due to the FQ-induced oxidative stress within the digestive vacuole and the subsequent destruction of the membrane resulting in death of the parasite [12].

The aim of the present work was to study the antischistosomal activities of FQ against *S. mansoni in vitro* and *in vivo*. In addition, we investigated whether redox activation plays a role in antischistosomal activity by including the ruthenocenyl analogue of FQ, namely ruthenoquine (RQ, Figure 1), which was shown, contrary to FQ, to be unable to produce reactive oxygen species (ROS) in *P. falciparum* [13]. This difference in redox activity was assumed to be responsible for the greater antimalarial activity of FQ over RQ. Furthermore, the antischistosomal activity of hydroxyl-ferroquine (FQ-OH, Figure 1) was also studied since FQ-OH is also able to produce hydroxyl radicals and provides reduced cytotoxic effects compared to FQ [14].

## Findings

### Methods

#### Animals and parasites

*In vivo* studies were carried out in accordance with Swiss national and cantonal regulations on animal welfare (permission no. 2070) at the Swiss Tropical and Public Health Institute (Basel, Switzerland). Female mice (NMRI strain, n = 31; weight ~ 20–22 g) were purchased from Charles River, Germany, kept under environmentally-controlled conditions (temperature ~ 25°C; humidity ~70%; 12-hour light and 12-hour dark cycle) with free access to water and rodent diet and acclimatized for one week before infection. Cercariae of *S. mansoni* were obtained from infected intermediate host snails (*Biomphalaria glabrata*) as described previously [15].

### Compounds

FQ, FQ-OH and RQ were synthesized according to reported procedures [13, 14, 16]. MQ was kindly obtained from Mepha AG (Aesch, Switzerland). CQ was purchased from Sigma (Buchs, Switzerland). For *in vitro* studies, compounds were dissolved in DMSO (Fluka, Buchs, Switzerland) to obtain stock solutions of 10 mg/ml. For *in vivo* studies, compounds were suspended in 7% (v/v) Tween 80 and 3% (v/v) ethanol shortly before oral treatment (10 ml/kg) of mice.

### *In vitro*studies

#### Newly transformed schistosomula (NTS)

*S. mansoni* cercariae were mechanically transformed to newly transformed schistosomula (NTS) [17]. A NTS suspension at a concentration of 100 NTS per 50 µl was prepared using Medium 199 (Invitrogen, Carlsbad, CA) [supplemented with 5% inactivated fetal calf serum (iFCS) and 100 U/ml penicillin and 100 mg/mL streptomycin (Invitrogen). NTS were incubated with 10 µM and 33.3 µM of the test compounds for 72 h. Compounds were tested at least in triplicate and the highest concentration of DMSO served as control. Plates were incubated at 37°C, 5% CO_2_. NTS were evaluated by microscopic readout (Carl Zeiss, Germany, magnification 80x) using a viability scale scoring death, changes in motility, viability, and morphological alterations [17].

### Adult *S. mansoni*

Adult schistosomes obtained from infected mice were incubated in the presence of 10 µM and 33.3 µM of the test compounds for up to 72 h. Phenotypes were monitored daily based on motility, viability and morphological alterations under an inverse microscope (Carl Zeiss, Germany, magnification 80×).

### Cytotoxicity studies

Cytotoxicity studies were performed on human cervix HeLa cancer cells and non tumorigenic human fetal lung fibroblasts MRC-5 to compare the activity of FQ, RQ, FQ-OH, CQ, MQ and cisplatin. The cell viability was determined via a colorimetric cell-based assay using Resazurin (Promocell GmbH). Briefly, one day before treatment cells were plated in triplicates in 96-well plates at a density of 4 × 10^3^ cells/well in 100 µl. Upon treating cells with increasing concentrations of the target complexes (freshly prepared stock solution in DMSO), cells were incubated at 37°C/6% CO_2_ for 48 h, the medium was removed, and 100 µl of complete medium containing resazurin (0.2 mg/ml final concentration) was added. After 4 h of incubation at 37°C/6% CO_2_, the fluorescence of the highly red fluorescent resorufin product was quantified at 590 nm emission with 540 nm excitation wavelength in a SpectraMax M5 microplate Reader.

### *S. mansoni in vivo*studies

Groups of 3–4 NMRI mice were treated orally with single oral doses of 200 mg/kg of FQ, FQ-OH and RQ. In addition, one group of mice was treated with a single oral dose of 800 mg/kg FQ. Untreated mice served as controls in all experiments. At 21 d post-treatment, animals were killed by the CO_2_ method and dissected. Worms were removed by picking, then sexed and counted as previously described [18].

### Statistics

Parasite viability values of treated and untreated worms obtained from microscopic evaluation were averaged (means (+/− standard deviation)) using Microsoft Excel software. The Kruskal-Wallis test was applied for *in vivo* studies, comparing the medians of the worm counts of the treatment and control groups. A difference in median was considered to be significant at a significance level of 5% (StatsDirect statistical software, version 2.7.2.; StatsDirect Ltd., United Kingdom).

## Results and discussion

### Cytotoxicity studies

We assessed the potential toxicity of the compounds studied in this work on cervical cancer cells (HeLa) and non-cancerous cells (MRC-5). Cisplatin, the best known metal-based drug on the market was used as a reference. As shown in Table 1, all metal complexes were shown to be moderately cytotoxic. MQ was the most toxic compound in these assays. This is not surprising, since MQ is well known for its adverse events, including gastrointestinal effects [9] and neuropsychiatric toxicity [19]. On the other hand, FQ was well tolerated and shown to be lacking relevant adverse effects on central nervous system, respiratory, renal, and gastrointestinal functions in a phase I trial [20]. However, larger trials are necessary to confirm this finding.

**Table 1:** **IC**_**50**_ **values for ferroquine (FQ), hydroxyl-ferroquine (FQ-OH), ruthenoquine (RQ), chloroquine (CQ) and mefloquine (MQ) in non-cancerous MRC-5 and HeLa cancer cells**

Complex	IC _50_ MRC-5 (µM)	IC _50_ HeLa (µM)
Cisplatin	7.9 ^ ± 1.2^	11.5 ^ ± 2.9^
FQ	24.4 ^ ± 0.9^	10.1 ^ ± 0.3^
FQ-OH	22.6 ^ ± 1.2^	16.8 ^ ± 1.5^
RQ	21.9 ^ ± 2.6^	8.8 ^ ± 0.41^
CQ	55.1 ^ ± 1.3^	87.0 ^ ± 1.5^
MQ	16.7 ^ ± 0.2^	6.7 ^ ± 0.7^

Cisplatin was used as positive control. Standard deviations are shown in superscript.

### Activity against NTS and adult *S. mansoni in vitro*

**Figure 2:**
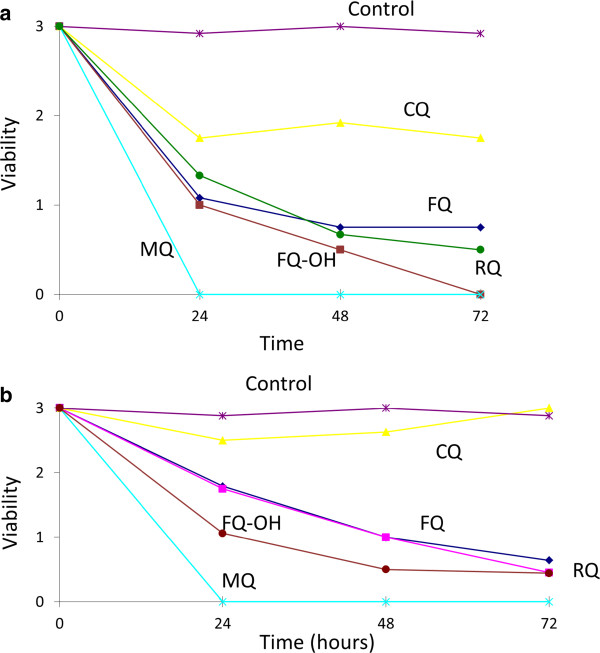
**Activity of ferroquine (FQ), hydroxy-ferroquine (FQ-OH), ruthenoquine (RQ), chloroquine (CQ) and mefloquine (MQ) versus untreated control worms in vitro at 33.3** **µg/ml against NTS (a) and adult** ***S. mansoni*** **(b).**

24 hours post-incubation all NTS exposed to 33.3 µM FQ, FQ-OH and RQ showed strongly reduced viabilities. 72 hours post-incubation all NTS exposed to 33.3 µM RQ had died, while FQ and FQ-OH treated worms were strongly affected but still alive. Therefore, unlike for *P. falciparum* and *Trypanosoma brucei gambiense* [21], oxidative shock does not seem to play a role in the activity of these compounds against *S. mansoni*, since RQ, which cannot produce ROS is the most active of the three organometallic drug against NTS *in vitro*. This absence of redox activation could be explained by the difference in the target of FQ, FQ-OH and RQ in *S. mansoni* compared to *P. falciparum.* In *P. falciparum*, sour conditions present in the acidic compartment where FQ localizes as well as of H_2_O_2_ are necessary for the formation of HO^**.**^ [12]. For comparison, no activity was observed treating NTS with CQ at 33.3 µM (Figure 2a) while at this concentration 24 hours post-incubation with MQ all worms had died. The antischistosomal mechanism of action of MQ and CQ is also not known. Note that these 2 drugs do not produce hydroxyl radicals [22]. The antimalarial activity of CQ and MQ is largely attributed to an inhibition of hemoglobin degradation. However, it was recently shown that hemozoin inhibition of CQ, MQ or quinine does not exhibit a correlation with their antischistosomal properties [23]. Furthermore, it was demonstrated that MQ interferes with glycolysis in NTS [24].

Exposure to 10 µM of the test drugs resulted in reduced motilities of NTS but the larvae did not die (data not shown).

A similar trend was observed on adult *S. mansoni*. Incubation of adult *S. mansoni* with 33.3 µM of MQ resulted in death of worms 24 h post-treatment, while adult *S. mansoni* exposed to the organometallic derivatives were highly affected in viability but were still alive 72 hours post-incubation (Figure 2b). At 10 µM adults showed strongly reduced viabilities 48–72 hours post-incubation with all derivatives (data not shown).

### Activity against adult *S. mansoni in vivo*

Compounds progressed into *in vivo* studies despite the fact that *in vitro* activities were only observed at concentrations that were close to cytotoxic concentrations. However, in our recent studies MQ was well tolerated by mice [8].

We have previously shown that CQ lacked antischistosomal activity *in vivo* (worm burden reduction of 11%) [8]. FQ is known for important and additional properties (e.g. its higher lipophilicity) and mechanism of actions (the above mentioned radical formation as well as hemozoin inhibition) compared to CQ [22]. Yet, treatment of mice with 200 and 800 mg/kg FQ, showed low total worm burden reductions of 19.4% and 35.6% (Table 2). One of the mice treated with 800 mg/kg FQ died within 24 hours post-treatment. No activity was observed treating mice with RQ at 200 mg/kg. Finally, a total worm burden reduction of 17.3% was observed following treatment with FQ-OH. Hence, modification of CQ by a ferrocenyl or ruthenocenyl fragment does not increase the antischistosomal properties of CQ. For comparison, at 200 mg/kg MQ achieved a much higher worm burden reduction of 72.3% in *S. mansoni*-infected mice [8]. A few issues of our *in vivo* results are worth highlighting: interestingly, a moderate female worm burden reduction of 43.7% (p = 0.018) was observed using FQ at 800 mg/kg (which had not been observed with CQ). A higher effect against female adult *S. mansoni* was also observed in MQ treated mice [8] pointing to a sex-specific interference of these drugs with the target. Furthermore, in one of the FQ-OH treated mice many dead worms were recovered and a hepatic shift (i.e. worms migrating to the liver) observed. Hence, FQ and FQ-OH show weak antischistosomal activity *in vivo*, which is in line with our *in vitro* results.

**Table 2:** **Effect on worm burden of single oral doses of three selected organometallic CQ derivatives administered to mice harboring a 49-day-old adult** ***S. mansoni*** **infection, stratified by sex and worm distribution**

Drug	Dose (mg/kg)	No. of mice investigated	No. of mice cured	Mean number of worms (SD)					Total worm burden reduction (%)	***p***-value	Female worm burden reduction (%)	***p***-value
				Liver	Mesenteric veins	Total	Males	Females				
Control^1^	-	8	-	0.4 (0.7)	33.8 (10.2)	34.1 (10.3)	19.9 (7.7)	14.3 (4.2)	-	-	-	-
Control^2^	-	8	-	0.6 (1.2)	25.8 (16.7)	26.4 (16.7)	14.5 (9.3)	11.9 (7.7)	-	-	-	-
FQ^a^	200	4	0	1.8 (2.4)	25.8 (7.3)	27.5 (7.3)	16.3 (4.9)	11.3 (4.0)	19.4	>0.05	21.0	>0.05
FQ^b^	800	4*	0	0.7 (1.2)	16.3 (4.2)	17.0 (5.3)	10.3 (5.8)	6.7 (1.2)	35.6	>0.05	43.7	0.018
FQ-OH^a,b^	200	3	0	3.7 (5.5)	21.3 (26.6)	25.0 (24.4)	16.3 (14.3)	8.7 (10.8)	17.3	>0.05	33.6	>0.05
RQ^a^	200	4	0	0.25 (0.5)	36.5 (10.7)	36.8 (10.4)	18.8 (5.3)	18.0 (5.3)	0	>0.05	0	>0.05
MQ	200	5	0						72.3 [8]		100	
CQ	200	5	0						11.7 [8]		93.0	

*one mouse died within 24 hours post-treatment, ^a^worm burden reduction calculated *versus* control ^1^;^b^worm burden reduction calculated *versus* control ^2^SD= standard deviation.

## Conclusions

In conclusion, the organometallic compounds evaluated in this study show only weak antischistosomal properties *in vivo*. Hence, based on our findings an ancillary benefit on schistosomiasis as a result of treating *P. falciparum* infections with FQ is not expected. Furthermore, no correlation can be drawn between the antimalarial and antischistosomal activity of CQ analogues, which might hint to distinct mechanisms of actions. Despite the low activities of organometallic drugs tested against *S. mansoni* so far [25] further derivatives (e.g. organometallic derivatives of MQ) should be studied.
